# Case Report: Guillain–Barré Syndrome Associated With COVID-19

**DOI:** 10.3389/fneur.2021.678136

**Published:** 2021-06-22

**Authors:** Eman M. Khedr, Ahmed Shoyb, Khaled O. Mohamed, Ahmed A. Karim, Mostafa Saber

**Affiliations:** ^1^Department of Neurology and Psychiatry, Assiut University, Asyut, Egypt; ^2^Department of Neuropsychiatry, Aswan University, Aswan, Egypt; ^3^Department of Psychiatry and Psychotherapy, University of Tübingen, Tübingen, Germany; ^4^Department of Health Psychology and Neurorehabilitation, SRH Mobile University, Riedlingen, Germany

**Keywords:** COVID-19, SARS-CoV-2, neurological association, Guillain-Barré syndrome, acute inflammatory demyelinating polyradiculoneuropathy, axonal Guillain-Barré syndrome, peripheral neuropathy, polymerase-chain-reaction

## Abstract

Guillain–Barré syndrome (GBS) is a potentially fatal, immune-mediated disease of the peripheral nervous system that is usually triggered by infection. Only a small number of cases of GBS associated with COVID-19 infection have been published. We report here five patients with GBS admitted to the Neurology, Psychiatry, and Neurosurgery Hospital, Assiut University/Egypt from July 1 to November 20, 2020. Three of the five patients were positive for SARS-CoV-2 following polymerase chain reaction (PCR) of nasopharyngeal swabs on day of admission and another one had a high level of IgM and IgG; all had bilateral ground-glass opacities with consolidation on CT chest scan (GGO) and lymphopenia. All patients presented with two or more of the following: fever, cough, malaise, vomiting, and diarrhea with variable duration. However, there were some peculiarities in the clinical presentation. First, there were only 3 to 14 days between the onset of COVID-19 symptoms and the first symptoms of GBS, which developed into flaccid areflexic quadriplegia with glove and stocking hypoesthesia. The second peculiarity was that three of the cases had cranial nerve involvement, suggesting that there may be a high incidence of cranial involvement in SARS-CoV-2-associated GBS. Other peculiarities occurred. Case 2 presented with a cerebellar hemorrhage before symptoms of COVID-19 and had a cardiac attack with elevated cardiac enzymes following onset of GBS symptoms. Case 5 was also unusual in that the onset began with bilateral facial palsy, which preceded the sensory and motor manifestations of GBS (descending course). Neurophysiological studies showed evidence of sensorimotor demyelinating polyradiculoneuropathy, suggesting acute inflammatory polyneuropathy (AIDP) in all patients. Three patients received plasmapheresis. All of them had either full recovery or partial recovery. Possible pathophysiological links between GBS and COVID-19 are discussed.

## Introduction

The first cases of COVID-19 reported from Wuhan, China occurred in December 2019, and on March 11, 2020, the World Health Organization (WHO) declared the novel coronavirus (COVID-19) outbreak a global pandemic. COVID-19 can produce a wide spectrum of central and peripheral nervous system disorders. The latter can include anosmia, ageusia, visual impairment, Guillain–Barré syndrome (GBS) (1–3), and skeletal muscle injury (3). GBS has been recorded after infection with Middle East respiratory syndrome (MERS) virus (4), and recently, a few case reports/series have noted that GBS can be associated with SARS-CoV-2 infection. GBS is an acute immune-mediated polyradiculo-neuropathy that may be triggered by various bacterial and viral infections. Approximately 70% of patients report a recent preceding upper or lower respiratory tract infection or gastrointestinal illness (5). Patients with GBS classically present with progressive symmetrical weakness and numbness of lower limbs that progresses to the upper limbs and is associated with hyporeflexia or areflexia. In severe cases, it can involve respiratory muscles and warrant use of mechanical ventilation (6). Disease progression can be rapid, and most patients with GBS reach their maximum disability within 2 weeks.

The first reported case of GBS associated with COVID-19 infection was of a woman who had just returned from Wuhan on January 19, 2020 (2). A recent review of published cases included 61 patients diagnosed with GBS and laboratory-confirmed preceding/concomitant SARS-CoV-2 (7). Most of the articles included were from high- and upper-middle-income countries according to the World Bank classification. Only one article was reported from Morocco, a lower-middle-income country in Africa.

Here, for the first time in Egypt, we discuss five cases of GBS associated with COVID-19 infection, admitted to the Neurology, Psychiatry, and Neurosurgery Hospital, Assiut University/Egypt during the period from July 1 to November 20, 2020. Clinical and laboratory data are presented in Table 1 and neurophysiological data are shown in Table 2.

**Table 1:** Clinical and laboratory data.

**Case**	**Age and sex**	**COVID-19 related symptoms**	**Clinical symptoms and signs of GBS MRC at admission and time evolved**	**EGRIS**	**Laboratory findings**	**PCR**	**CT Chest**	**Co-morbidities**	**Treatment and Outcome**
1	34 Male	10 days of fever, cough, expectoration	Flaccid areflexic quadriplegiay, stock and glove, hypothesia, bulbar palsy, respiratory failure → mechanical ventilator. MRC sum score: 0. The symptoms evolved over a week.	6	↑D-dimer Leukocytosis, Anemia, Lymphopenia	–ve	Bilateral GGO	None	Five sessions plasmapheresis → no improvement. 1 month later → 5 days IVIG 2 weeks later → weaned from ventilator MRC sum score on discharge: 12 Two months after discharge MRC sum score improved to be 34
2	65 Male	5 days of fever, malaise, cough	Flaccid areflexic quadriplegia, stock & glove hypothesia. MRC sum score: 12. The symptoms evolved over 2 days.	14	Neutropenia, Lymphopenia, ↓ PaCo2 & PaO2	+ve	Bilateral GGO	IHD, Cerebellar hemorrhage	5 sessions of plasmapheresis → improvement MRC sum score on discharge: 48
3	49 Female	3 days of fever and repeated vomiting	Flaccid areflexic quadriplegia, stock & glove hypothesia, Lt LMNL facial palsy, bilateral bulbar palsy, MRC sum score: 24 The symptoms evolved over 2 days.	5	↑D-dimer, Thrombocytosis, Lymphopenia ↓ PaCO2	+ve	Bilateral GGO	None	1 session of plasmapheresis → hypersensitivity reaction. 4 weeks later → 5 days IVIG. 1 week later → started to improve with MRC sum score on discharge: 36. Three weeks after discharge → she can walk with moderate support, MRC sum score 50
4	45 Male	14 days of fever, cough, diarrhea	Flaccid areflexic quadriparesis, stock & glove hypothesia. MRC sum score: 40. The symptoms evolved over 1 day.	4	Anemia, Thrombocytosis, Neutrophilia, Lymphopenia	+ve	Bilateral GGO	DM	Steroids for 2 weeks → marked improvement MRC sum score on discharge: 60 but he still complains from paresethesia of fingers and toes
5	55 Female	14 days of fever, cough, expectoration	Bilateral LMN facial palsy, followed by flaccid areflexic quadriplegia with weakness proximal more than distal, as well as stock and glove hypothesia. MRC sum score: 34. The symptoms evolved over 10 days.	4	Leukocytosis, Neutrophilia, Lymphopenia.	Unavailable High IgG and IgM	Bilateral GGO	None	Received 5 days of IVIG, and showed improvement on discharge that the patient walk with moderate support (MRC = 48). Two months later patient walk without support with the MRC sum score was 60

*EGRIS, Erasmus GBS Respiratory Insufficiency Score RF, respiratory failure; LMNL, lower motor neuron lesion; MRC, Medical Research Council; Lap, laboratory results; PaCo2, partial pressure of carbon dioxide; PaO2, partial pressure of oxygen; PCR, Polymerase chain reaction from nasopharyngeal swap; -ve, negative; +ve, positive; CT, computerized tomography; GGO, Ground-glass Opacity; AIDP, acute inflammatory demyelinating polyneuropathy; IHD, Ischemic heart disease; DM, Diabetes mellitus; IVIG, Intravenous immunoglobulin. ↑, increase; ↓, decrease*.

**Table 2:** Neurophysiological data.

	**Case 1**		**Case 2**		**Case 3**		**Case 4**		**Case 5**	
	**Rt**	**Lt**	**Rt**	**Lt**	**Rt**	**Lt**	**Rt**	**Lt**	**Rt**	**Lt**
**Median nerve**										
Motor DL (ms)	5.40	5.25	3.24	3.12	8.3	7.55	4.9	5.1	4.2	4.3
CMAP amplitude (mV)	9.45	10.3	11.05	9.57	1.76	1.67	17.8	18.7	1.45	1.01
MCV (m/s)	50.0	49.9	59.8	63.7	43.1	51.8	46.7	45.5	55.6	56.5
Sensory DL (ms)	NR	NR	2.46	2.52	NR	NR	NR	NR	3.54	3.64
Sensory amplitude (μV)	NR	NR	32.2	28.2	NR	NR	NR	NR	17.8	18.4
SCV (m/S)	NR	NR	56.9	55.6	NR	NR	NR	NR	39.4	40.5
F-wave (ms)	33.4	34.15	26.6	27.3	35.1	39.5	33.9	34.6	38.4	39.7
**Ulnar nerve**										
Motor DL (ms)	3.6	3.3	2.07	3.12	8.12	7.55	4.8	5.1	4.6	4.3
CMAP amplitude (mV)	1.4	1.6	7.59	9.57	1.54	1.67	19.5	18.7	1.54	1.01
MCV (m/s)	34.4	44	53.3	63.7	50.8	51.1	43.2	45.5	55.7	56.5
Sensory DL (ms)	3.34	3.42	2.52	3	NR	NR	NR	NR	3.74	3.46
Sensory amplitude (uV)	0.8	1	28.2	31	NR	NR	NR	NR	17.6	18.4
SCV (m/S)	38.6	40.9	55.6	52	NR	NR	NR	NR	38.7	40.5
F-wave (ms)	30.44	31.25	27.3	27.6	40	39	36.4	37	31.4	32.7
**Tibial nerve**										
Motor DL (ms)	6.7	6.9	6.15	8	9.75	7.15	5.3	5.6	NR	NR
CMAP amplitude (mV)	0.45	0.82	4.71	0.47	1.04	1.52	6.24	5.36	NR	NR
MCV (m/s)	40.4	39.5	43.1	73.4	51	31.7	33.2	31.5	NR	NR
F-wave (ms)	37.6	36.7	33.5	34.4	NR	34	38.5	38.5	NR	NR
**Peroneal nerve**										
Motor DL (ms)	7.2	7.6	3.05	4.3	6.4	5.75	4.5	4.7	10.5	11.9
CMAP amplitude (mV)	2.51	2.49	0.79	0.77	6.6	6.58	2.23	2.19	0.56	0.45
MCV (m/s)	45.7	44.6	48.2	48.5	39.1	39.8	30.4	38.3	38.1	39.3
F-wave (ms)	NR	NR	41.4	35.7	NR	NR	41.75	40.85	NR	NR

*DL, distal latency; MCV, motor conduction velocity; SCV, sensory conduction velocity; NR, no response*.

### Case 1

A 34-year-old male came to the emergency room with a history of cough, expectoration, and fever of 10 days duration followed by numbness of feet and then hands. On admission, polymerase chain reaction (PCR) of nasopharyngeal swab was negative, CT chest showed ground-glass opacities (GGO), and laboratory investigations (↑D-dimer, leukocytosis, anemia, and lymphopenia) suggested probable COVID-19 infection. During admission, he developed weakness of both distal and proximal lower and then upper limbs that progressed rapidly until he became bedridden 1 week later. Two days after this, he developed dysphagia and a nasal tone of speech. The details of the clinical examination, Erasmus GBS Respiratory Insufficiency Score (EGRIS), the medical Research Council (MRC) sum score, the positive laboratory data, and the result of CT chest at the day of admission are illustrated in Table 1. The patient was connected to mechanical ventilation.

Neurophysiological study showed evidence of mixed demyelinating and axonal variant of GBS (AIDP); detailed measures are in Table 2.

Due to unavailability of intravenous immunoglobulin (IVIG) at that time, the patient received five sessions of plasma exchange (200–250 ml plasma/kg body weight) with no improvement either in motor or in respiratory functions. One month later, the patient received a course of IVIG (0.4 g/kg body weight daily for 5 days). Two weeks later, the patient was weaned from the ventilator and discharged home with motor power grade 1 in proximal and distal muscles of both upper and lower limbs. MRC sum score was 12. Follow-up 2 months after discharge, the patient MRC sum score had increased to 34.

### Case 2

A 65-year-old male came to the emergency room with a history of left hemiataxia of 10 days duration; CT brain showed left cerebellar hemorrhage. He received medical treatment and discharged to his home with mild improvement but required mild support to walk. One week later, the patient developed fever, malaise, and cough for 2 days and readmitted again to the hospital and positive PCR was recorded, and then he developed numbness and weakness of all four limbs and within 2 days became bedridden. Details of clinical examinations, EGRIS, MRC sum score, and laboratory findings on admission are shown in Table 1. On the third day of admission, the patient had a cardiac attack with elevated liver enzymes for which he received medical treatment and improved.

Blood picture was normal except for the presence of lymphopenia and neutropenia. ABG showed low PaO2 and PaCo2. CT chest showed GGO.

A neurophysiological study showed evidence of a mixed axonal and demyelinating variant of GBS (AIDP); detailed measures are in Table 2.

The patient received five sessions of plasmapheresis (one session every other day) and showed marked improvement in motor power such that he could walk with mild support on discharge. MRC sum score was 48.

### Case 3

A 49-year-old female had a history of fever and repeated vomiting for 3 days followed by numbness of hands and feet at the time she admitted to the hospital and positive PCR of nasopharyngeal swabs and bilateral GGO of CT chest were recorded; 1 day later, the patient developed rapid progressive symmetrical weakness of both lower and upper limbs, proximal more than distal, and within 2 days, she became bedridden (rapidly evolving). On examination, the patient was fully conscious, with intact cranial nerves. Motor examination showed hypotonia of both upper and lower limbs, reduced muscle strength (MRC grades were 3 for proximal and 4a for distal muscles of both upper limbs; 0 for proximal and 1 for distal muscles of both lower limbs), with four limbs. Sensory examination revealed a glove and stocking hypoesthesia. Three days after admission, the patient developed deviation of the angle of the mouth to the right side and was unable to close her left eye, dysphagia, hoarseness of voice, and an impaired cough reflex. On examination, there was a left lower motor neuron facial palsy and true bulbar palsy with reduced gag reflex. The EGRIS was 5 and the MRC sum score was 24. Details of laboratory findings are in Table 1. ABG showed low PaCO2. Otherwise, PT, PC, INR, liver, and renal functions were normal. All electrolytes were normal (Na^+^, K^+^, and Ca^2+^).

A neurophysiological study provided evidence of a mixed axonal and demyelinating variant of GBS. Detailed data are given in Table 2.

Ten days after admission, the patient received the first session of plasmapheresis, during which she showed a hypersensitivity reaction with respiratory failure. She was admitted to the ICU with an oxygen mask and 40% ventilation. Nasopharyngeal swabs were repeated three times on a weekly basis (firstly at admission) and were positive for COVID-19; the fourth swab was negative. Four weeks later, the patient received 5 days of IVIG, and after 1 week, the motor power of both upper and lower limbs was improved. The MRC sum score was 36. Three weeks after discharge, she could walk with moderate support and had an MRC sum score of 50.

### Case 4

A 45-year-old male, known to be diabetic, came to the emergency room complaining of malaise, fatigability, nausea, and vomiting associated with diffuse abdominal pain. A diagnosis of diabetic ketoacidosis was made, and he was admitted for management. Two days later, the patient developed fever, cough, and diarrhea. Chest CT showed bilateral GGO, and he tested positive for COVID-19 following a RT-PCR from a nasopharyngeal swab. The patient was transferred to the isolation hospital for COVID-19 patients (Abo Teg isolation hospital) where he stayed for 5 days and then was discharged home. One week after discharge, the patient complained of paresthesia of the feet and hands, and within a day, he developed bilateral symmetrical weakness of both lower and upper limbs, particularly in proximal muscles. Data of the clinical examination, EGRIS, MRC sum score, laboratory data, and CT chest on admission are given in Table 1.

A neurophysiological study showed evidence of a demyelinating variant of GBS. Detailed data are given in Table 2.

The patient received steroids for 2 weeks and showed marked improvement. The patient was discharged 2 weeks later with a motor power of 5 proximally and distally of both upper and lower limbs. The MRC sum score was 60, but the patient still complains of paresthesia.

### Case 5

A 55-year-old female came to the emergency room with a 2-week history of low-grade fever, cough, and expectoration followed by progressive bilateral facial weakness. Because of the unavailability of PCR of nasopharyngeal swab, CT chest showed bilateral GGO and laboratory investigations were done, revealing leukocytosis, neutrophilia, and lymphopenia (probable COVID-19). On examination, the patient was fully conscious and was unable to close both her eyes, with reduced blinking, inability to whistle, protrude the lips, or expose the teeth. One week later, after the onset of facial weakness, she developed numbness of both hands and feet, associated with progressive symmetrical weakness of both lower and upper limbs, affecting proximal more than distal muscles and was bedridden by the end of the week. Clinical details, EGRIS, MRC sum score, and CT chest are given in Table 1.

Titers of antibodies (IgG and IgM) against the SARS-CoV-2 were elevated 3 weeks after the onset of COVID-19. Table 1 gives details of laboratory investigations.

Neurophysiological investigation revealed a mixed axonal and demyelinating variant of GBS. Detailed data are given in Table 2.

The patient received 5 days of IVIG, after which she started to walk with moderate support after 1 week. Two weeks later, she walked with mild support, and on discharge, the MRC sum score was 48. Two months later, she was walking without support with an MRC sum score of 60.

## Discussion

We recruited five cases of GBS-associated COVID-19 over the period from July 1 to November 20, 2020. Three were males and two were female with a mean age of 49.6 ± 11.52 years ranging from 34 to 65. Three of them were younger than 50 years old. In a recent systematic review, Hasan et al. (7) found that of 61 GBS cases associated with COVID-19, two-thirds (68.9%) were male and had a median age of 57 (49–70) years (7). So far, only three children < 18 years have been diagnosed with GBS following a coronavirus infection (8–10).

All our patients had symptoms including fever, malaise, headache, respiratory tract symptoms, and GIT symptoms. None had lost smell or taste. PCR of nasopharyngeal swabs was confirmed positive in three patients and IgM and IgG for corona virus were high in one patient. We did not test for SARS-CoV-2 in CSF since no cerebrospinal fluid samples have tested positive for COVID-19 in any reported cases summarized in systematic reviews of COVID-19 and GBS (11, 12). All patients had decreased lymphocytic count and CT chest with characteristic bilateral (GGO).

In the present study the peculiarities of our cases included the short latent period between the onset of COVID-19 and onset of GBS symptoms (7.2 ± 4.6 days with a range from 3 to 14 days), which is shorter than that reported in the systematic review of Hasan et al. (7). They reported that the median interval between SARS-CoV-2 symptoms and onset of GBS was 14 days with a range of 2 to 33 days (7). The slightly shorter duration in our cases could be explained by an asymptomatic incubation period, denial of early symptoms of COVID-19, or a hyper-inflammatory response as previously suggested by Zhao et al. (2). A similar result was reported by Kajumba et al. (12) in their review of 51 cases of GBS associated with COVID-19: that is, there was only a short time interval between the onset of the COVID-19 and GBS symptoms in comparison to the classic type of GBS. In our study, there was not only a rapid onset of symptoms but also rapid progression (4.4 ± 3.5 days) compared with classic GBS patients who typically reach maximum disability within 2 weeks (13).

The first reported case of GBS linked to COVID-19 was a woman who had returned from Wuhan (the presumed epicenter of the pandemic). She presented with weakness of both lower and both upper limbs and distal hypoesthesia, but had no signs or symptoms of COVID-19 until the eighth day when she developed dry cough and low-grade fever; a PCR was positive for SARS-CoV-2 (2). GBS in this first case followed the pattern of a para-infectious profile similar to GBS associated with Zika virus (14), rather than the classic post-infectious profile of most COVID-19 cases. The time elapsed between the onset of COVID-19 manifestations and GBS symptoms provides clues to the pathogenesis of GBS in SARS-CoV-2. We assume a post-infectious mechanism in the present study likely mediated by immune-mediated damage to peripheral nerve (15, 16). This could be the result of the viral spike protein binding to the receptor of the angiotensin-converting enzyme 2 (17). Alternatively, it could involve T-cell activation and release of inflammatory mediators by macrophages. A recent systematic review found that none of the patients had positive PCR for SARS-CoV-2 in the CSF (18). The absence of evidence for active infection when patients have clinical symptoms of GBS makes an immune-mediated mechanism the most likely cause of GBS associated with SARS-CoV-2.

In the present study, all five patients fulfilled level 2 of the Brighton criteria (6) manifesting with bilateral, flaccid weakness and areflexia in all four limbs. They had a monophasic course, with neurophysiological evidence of AIDP. However, there were some peculiarities in the clinical presentation as three of five cases had cranial nerve affections, suggesting a potential for more cranial nerve involvement of SARS-CoV-2-associated GBS as compared with GBS associated with other infection.

There were other peculiarities in the presentation of cases 2 and 5. Case 2 presented with a cerebellar hemorrhage before symptoms of COVID-19 and had a cardiac attack with elevated cardiac enzymes following onset of GBS symptoms. The association of hemorrhagic strokes with COVID-19 could be related to fluctuations in blood pressure caused by the affinity of SARS-CoV-2 for ACE2 receptors, which are expressed in endothelial and arterial smooth muscle cells in the brain, which, in turn, leads to damage to intracranial blood vessels and wall rupture (19). The same mechanism is probably responsible for myocardial injury, arrhythmia, and acute coronary syndrome that can be associated with SARS-CoV-2 (20).

Case 5 was also unusual in that the onset was associated with bilateral facial palsy that preceded the sensory and motor manifestations of GBS. This is a rare form of GBS as the classic form started usually with the ascending form of acute progressive tetraplegia and simultaneous bilateral facial palsy (21). However, Caamaño and Beato (22) and Chan et al. (23) recorded a case report of facial diplegia associated with COVID-19 but without limb weakness. These different varieties of GBS-associated COVID-19 may be related to the pattern of invasion of the virus to the peripheral nerves.

An important limitation of this case was the elevated IgG and IgM 3 weeks after the onset of symptoms with the possibility to be related to either IVIG or SARS-CoV-2. However, IVIG has no direct effect on B cell proliferation and immunoglobulin production (24) and did not affect the detection or titers of IgG and IgM. On the other hand, the proportion of IgM-positive sera from the COVID-19 patients was maximal at 83% before 10 days and decreased to 0% after 100 days, while IgG-positive sera tended to plateau between days 11 and 65 at 78–100% and fall to 44% after 100 days (25). So, the elevated IgG and IgM in case 5 might be related to SARS-CoV-2.

Cases 1 and 4 had an uncommon response to treatment. Case 1 failed to respond to plasma exchange but later had a good response from IVIG, whereas IVIG and plasma exchange are usually of similar efficacy in classic GBS (26). In case 4, there was a marked improvement of motor powers after steroid therapy. Although corticosteroids would be expected to be beneficial in reducing inflammation, eight randomized controlled trials on the efficacy of corticosteroids for GBS showed no significant benefit and found that patients treated with oral corticosteroids have poor outcome (27). The positive effect in our cases could be due to the fact that corticosteroids were beneficial in reducing inflammation or were immunomodulatory in patients with COVID-19.

According to neurophysiological subtyping (28), all of our patients had neurophysiological evidence of AIDP. This is the most frequently reported variant of GBS in association with COVID-19 (18).

In the majority of previously published case reports, the outcomes were not clearly described. Here, we were able to present details of the clinical outcome of all five patients (see Table 1). All of them had either full recovery or partial recovery.

Although the GBS subtype diagnosis has currently no impact on treatment, we believe that it is important for understanding the underlying pathophysiology. Both IVIG and plasmapheresis are effective treatments for GBS with favorable outcome and a good adverse effect profile. IVIG is considered the first choice treatment because it is relatively easy to administer, is widely available, and has fewer side effects (29). One of our patients received corticosteroids with improvement in motor power within a month.

Early recognition and treatment with IVIG or plasma exchange/plasmapheresis (PLEX), along with supportive care, remains the mainstay of therapy (30–32).

Because we did not test the CSF for SARS-CoV-2 in our cases, there is no proof that COVID caused GBS. Nevertheless, the association between GBS and COVID needs further investigations.

In contrast to the presence of peculiarities of our cases, in a recent epidemiological and cohort study recorded by Keddie et al. (33), they compared COVID-19 (definite or probable)- and non-COVID-19-associated GBS patients and found no significant differences in the pattern of weakness, time to nadir, neurophysiology, or outcome. Two other systematic reviews by Abu-Rumeileh et al. (11) and Sansone et al. (34) showed that the clinical picture of COVID-19-associated GBS seems to resemble that of classic GBS or Zika virus-associated GBS.

Future epidemiological studies are required to evaluate the incidence rate of GBS in COVID-19. Moreover, studies combining treatment interventions with neurophysiological data are needed to investigate the underlying pathophysiological mechanisms.

## Data Availability Statement

The raw data supporting the conclusions of this article will be made available by the authors, without undue reservation.

## Ethics Statement

The studies involving human participants were reviewed and approved by Faculty of Medicine, Assiut University Hospital, Assiut, Egypt. The patients/participants provided their written informed consent to participate in this study.

## Author Contributions

EK: study conception, design of the work, statistical analysis, recruited the cases, critical revision of the manuscript, and prepared final version. KM, AS, and MS: recruited the cases, performed neurophysiological study, drafting the manuscript. AK: revised the manuscript and performed analysis. All authors gave final approval of the version to be published.

## Conflict of Interest

The authors declare that the research was conducted in the absence of any commercial or financial relationships that could be construed as a potential conflict of interest.
